# 1,1′-(Ethane-1,2-di­yl)bis­(4-{(*E*)-2-[4-(di­methyl­amino)­phen­yl]ethen­yl}pyridin-1-ium) dibromide ethanol 0.67-solvate

**DOI:** 10.1107/S2414314621003965

**Published:** 2021-06-04

**Authors:** Shengnan Wang, Gang Liu, Zhichao Wu

**Affiliations:** a Department of Chemistry, Anhui University, Hefei, Anhui 230039, People’s Republic of China; University of Aberdeen, Scotland

**Keywords:** crystal structure, pyridinium, bromide

## Abstract

The asymmetric unit of the title solvated mol­ecular salt, 3C_32_H_36_Br_2_N_4_^2+^·6Br^−^·2C_2_H_5_OH, consists of 1.5 cations, three bromide anions and one ethanol solvent mol­ecule of crystallization. The half-cation is completed by crystallographic inversion symmetry. In the crystal, O—H⋯Br hydrogen bonds and weak C—H⋯Br inter­actions link the components.

## Abstract 2

The asymmetric unit of the title solvated mol­ecular salt consists of 1.5 cations, three bromide anions and one ethanol solvent mol­ecule of crystallization. The half-cation is completed by crystallographic inversion symmetry. In the crystal, O—H⋯Br hydrogen bonds and weak C—H⋯Br inter­actions link the components.

## Graphical abstract

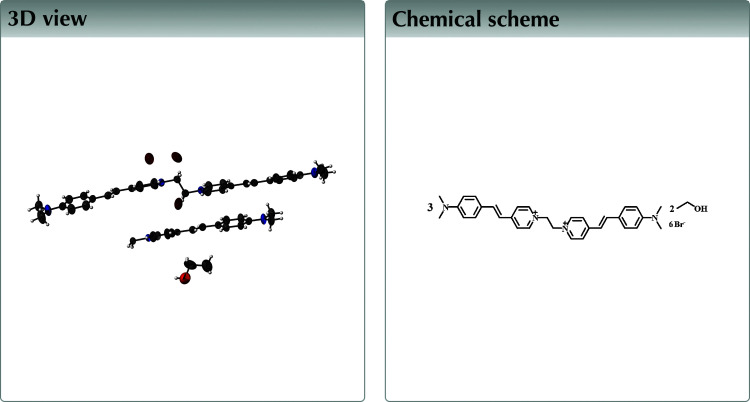

## Structure description

Double pyridine–bromide salts have been proposed as radical generators in cancer treatment (Bu *et al.*, 2020 ▸). We now describe the synthesis and structure of the title solvated mol­ecular salt, 3C_32_H_36_Br_2_N_4_^2+^·6Br^−^·2C_2_H_5_OH.

**Figure 1:**
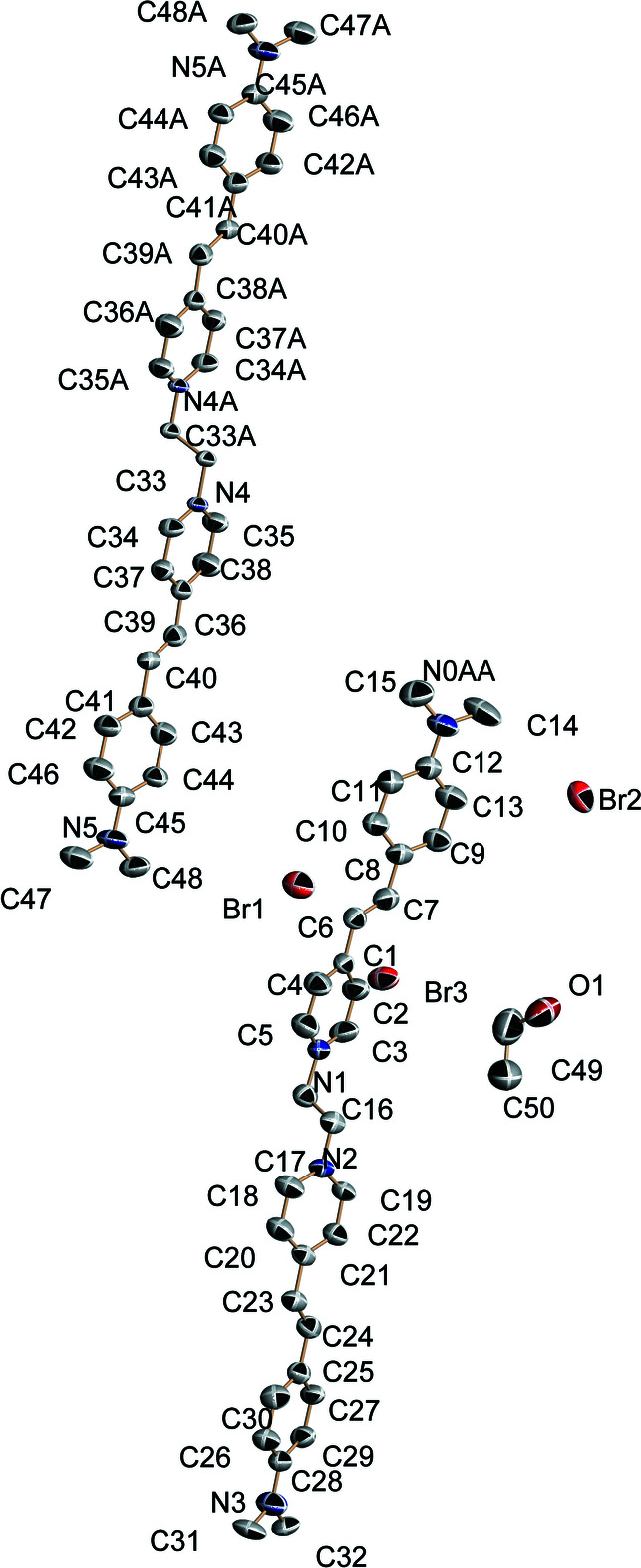
The mol­ecular structure of the title compound, with displacement ellipsoids drawn at the 30% probability level; H atoms are omitted for clarity.

The asymmetric unit consists of 1.5 cations, three bromide anions and one ethanol solvent mol­ecule of crystallization (Fig. 1 ▸), the half-cation being completed by crystallographic inversion symmetry. The dihedral angles between the N1/C1–C4 and N6/C18–22 rings is 15.2 (2)°.

In the crystal, O—H⋯Br hydrogen bonds and weak C—H⋯Br inter­actions link the components (Table 1 ▸). The shortest aromatic π–π stacking contact is 3.652 (3) Å between the centroids of the N6/C18–22 and C41–C46 rings.

## Synthesis and crystallization

4-Di­methyl­amino­benzaldehyde (0.44 g, 2.94 mmol), 1,1′-(ethane-1,2-di­yl)bis­(4-methyl­pyridin-1-ium) bromide (0.50 g, 1.33 mmol) and three drops of piperidine were dissolved in ethyl alcohol (40 ml). The mixture was heated to 80°C for 8 h, then cooled to room temperature. The crude product was recrystallized from mixed solvents (ethyl acetate/methanol = 4:7) as red blocks (0.71 g, 1.50 mmol).

## Refinement

Crystal data, data collection and structure refinement details are summarized in Table 2 ▸.

## Supplementary Material

Crystal structure: contains datablock(s) I. DOI: 10.1107/S2414314621003965/hb4370sup1.cif

Structure factors: contains datablock(s) I. DOI: 10.1107/S2414314621003965/hb4370Isup3.hkl

CCDC reference: 2077383

Additional supporting information:  crystallographic information; 3D view; checkCIF report

## Figures and Tables

**Table 1:** Hydrogen-bond geometry (Å, °)

*D*—H⋯*A*	*D*—H	H⋯*A*	*D*⋯*A*	*D*—H⋯*A*
O1—H1⋯Br1^i^	0.82	2.50	3.310 (6)	171
C16—H16*B*⋯Br2	0.97	2.92	3.841 (5)	158
C19—H19⋯Br3	0.93	2.87	3.765 (5)	162
C34—H34⋯Br3^ii^	0.93	2.85	3.761 (5)	167

Symmetry codes: (i) 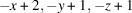 ; (ii) 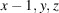 .

**Table 2:** Experimental details

Crystal data	
Chemical formula	3C_32_H_36_N_4_^2+^·6Br^−^·2C_2_H_6_O
*M*_r_	2001.53
Crystal system, space group	Triclinic, *P* 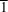
Temperature (K)	296
*a*, *b*, *c* (Å)	9.652 (3), 14.867 (6), 17.914 (6)
α, β, γ (°)	94.222 (7), 103.853 (5), 100.292 (4)
*V* (Å^3^)	2437.2 (15)
*Z*	1
Radiation type	Mo *K*α
μ (mm^−1^)	2.52
Crystal size (mm)	0.19 × 0.15 × 0.12

Data collection	
Diffractometer	Bruker SMART APEX CCD
Absorption correction	Multi-scan (*SADABS*; Bruker, 2013 ▸)
*T*_min_, *T*_max_	0.456, 0.746
No. of measured, independent and observed [*I* > 2σ(*I*)] reflections	17772, 8543, 4501
*R*_int_	0.057
(sin θ/λ)_max_ (Å^−1^)	0.595

Refinement	
*R*[*F* ^2^ > 2σ(*F* ^2^)], *wR*(*F* ^2^), *S*	0.052, 0.153, 0.96
No. of reflections	8543
No. of parameters	549
H-atom treatment	H-atom parameters constrained
Δρ_max_, Δρ_min_ (e Å^−3^)	0.53, −0.30

Computer programs: *APEX2* and *SAINT* (Bruker, 2013 ▸), *SHELXT* (Sheldrick, 2015*a* ▸), *SHELXL2014/7* (Sheldrick, 2015*b* ▸) and *OLEX2* (Dolomanov *et al.*, 2009 ▸).

## full crystallographic data

### Crystal data

**Table:**

3C_32_H_36_N_4_^2+^·6Br^−^·2C_2_H_6_O	*Z* = 1
*M**_r_* = 2001.53	*F*(000) = 1030
Triclinic, *P*1	*D*_x_ = 1.364 Mg m^−^^3^
*a* = 9.652 (3) Å	Mo *K*α radiation, λ = 0.71073 Å
*b* = 14.867 (6) Å	Cell parameters from 3435 reflections
*c* = 17.914 (6) Å	θ = 2.4–21.1°
α = 94.222 (7)°	µ = 2.52 mm^−^^1^
β = 103.853 (5)°	*T* = 296 K
γ = 100.292 (4)°	Block, red
*V* = 2437.2 (15) Å^3^	0.19 × 0.15 × 0.12 mm

### Data collection

**Table:**

Bruker SMART APEX CCD diffractometer	8543 independent reflections
Radiation source: sealed tube	4501 reflections with *I* > 2σ(*I*)
Graphite monochromator	*R*_int_ = 0.057
Detector resolution: 8 pixels mm^-1^	θ_max_ = 25.0°, θ_min_ = 1.7°
phi and ω scans	*h* = −11→11
Absorption correction: multi-scan (SADABS; Bruker, 2013)	*k* = −17→17
*T*_min_ = 0.456, *T*_max_ = 0.746	*l* = −21→21
17772 measured reflections	

### Refinement

**Table:**

Refinement on *F*^2^	Primary atom site location: dual
Least-squares matrix: full	Hydrogen site location: inferred from neighbouring sites
*R*[*F*^2^ > 2σ(*F*^2^)] = 0.052	H-atom parameters constrained
*wR*(*F*^2^) = 0.153	*w* = 1/[σ^2^(*F*_o_^2^) + (0.0713*P*)^2^] where *P* = (*F*_o_^2^ + 2*F*_c_^2^)/3
*S* = 0.96	(Δ/σ)_max_ = 0.001
8543 reflections	Δρ_max_ = 0.53 e Å^−^^3^
549 parameters	Δρ_min_ = −0.30 e Å^−^^3^
0 restraints	

### Special details

**Table:**

Geometry. All esds (except the esd in the dihedral angle between two l.s. planes) are estimated using the full covariance matrix. The cell esds are taken into account individually in the estimation of esds in distances, angles and torsion angles; correlations between esds in cell parameters are only used when they are defined by crystal symmetry. An approximate (isotropic) treatment of cell esds is used for estimating esds involving l.s. planes.

### Fractional atomic coordinates and isotropic or equivalent isotropic displacement parameters (Å^2^)

**Table:**

	*x*	*y*	*z*	*U*_iso_*/*U*_eq_	
Br1	1.13112 (6)	0.29512 (4)	0.27321 (4)	0.0854 (2)	
Br2	0.50470 (6)	−0.07151 (4)	0.37597 (4)	0.0820 (2)	
Br3	1.15643 (6)	0.35699 (4)	0.55618 (4)	0.0806 (2)	
O1	1.0330 (7)	0.6062 (4)	0.8733 (3)	0.1303 (18)	
H1	0.9961	0.6363	0.8402	0.195*	
C49	1.0734 (8)	0.5353 (5)	0.8390 (5)	0.123 (3)	
H49A	1.1410	0.5577	0.8090	0.148*	
H49B	0.9890	0.4944	0.8045	0.148*	
C50	1.1475 (8)	0.4838 (5)	0.9044 (4)	0.120 (3)	
H50A	1.1850	0.4354	0.8825	0.179*	
H50B	1.0774	0.4578	0.9310	0.179*	
H50C	1.2260	0.5262	0.9403	0.179*	
N1	0.7483 (4)	0.2030 (3)	0.4151 (2)	0.0515 (10)	
N2	0.3218 (5)	0.7345 (3)	0.1126 (3)	0.0759 (14)	
N3	1.6135 (4)	−0.2920 (3)	0.9295 (3)	0.0683 (12)	
N6	0.9098 (4)	0.0969 (3)	0.5900 (2)	0.0505 (10)	
C1	0.6117 (5)	0.3269 (3)	0.3307 (3)	0.0520 (12)	
C2	0.7631 (6)	0.3331 (4)	0.3485 (3)	0.0711 (16)	
H2	0.8204	0.3801	0.3312	0.085*	
C3	0.8288 (6)	0.2718 (4)	0.3907 (3)	0.0691 (15)	
H3	0.9295	0.2782	0.4024	0.083*	
C4	0.5337 (5)	0.2541 (3)	0.3578 (3)	0.0606 (14)	
H4	0.4329	0.2464	0.3476	0.073*	
C5	0.6025 (5)	0.1939 (3)	0.3991 (3)	0.0644 (15)	
H5	0.5478	0.1457	0.4164	0.077*	
C6	0.5360 (5)	0.3892 (3)	0.2860 (3)	0.0535 (13)	
H6	0.4358	0.3703	0.2658	0.064*	
C7	0.5971 (5)	0.4701 (3)	0.2716 (3)	0.0570 (13)	
H7	0.6978	0.4873	0.2905	0.068*	
C8	0.5247 (5)	0.5361 (3)	0.2292 (3)	0.0504 (12)	
C9	0.6003 (5)	0.6249 (4)	0.2298 (3)	0.0663 (15)	
H9	0.6980	0.6412	0.2569	0.080*	
C10	0.3785 (6)	0.5151 (3)	0.1871 (3)	0.0615 (14)	
H10	0.3237	0.4565	0.1856	0.074*	
C11	0.3139 (6)	0.5793 (3)	0.1479 (3)	0.0642 (15)	
H11	0.2172	0.5624	0.1192	0.077*	
C12	0.3894 (5)	0.6690 (3)	0.1498 (3)	0.0553 (13)	
C13	0.5350 (6)	0.6894 (4)	0.1916 (3)	0.0716 (16)	
H13	0.5897	0.7482	0.1937	0.086*	
C14	0.4026 (6)	0.8251 (4)	0.1136 (4)	0.105 (2)	
H14A	0.4590	0.8482	0.1654	0.157*	
H14B	0.3364	0.8648	0.0959	0.157*	
H14C	0.4665	0.8233	0.0801	0.157*	
C15	0.1759 (6)	0.7097 (4)	0.0636 (3)	0.0824 (18)	
H15A	0.1730	0.6661	0.0207	0.124*	
H15B	0.1460	0.7638	0.0446	0.124*	
H15C	0.1111	0.6827	0.0927	0.124*	
C16	0.8186 (5)	0.1359 (3)	0.4603 (3)	0.0534 (13)	
H16A	0.9156	0.1382	0.4532	0.064*	
H16B	0.7622	0.0740	0.4421	0.064*	
C17	0.8276 (5)	0.1588 (3)	0.5442 (3)	0.0544 (13)	
H17A	0.8765	0.2225	0.5613	0.065*	
H17B	0.7303	0.1511	0.5520	0.065*	
C18	0.8412 (5)	0.0155 (3)	0.6034 (3)	0.0628 (14)	
H18	0.7411	−0.0031	0.5826	0.075*	
C19	1.0551 (5)	0.1230 (3)	0.6184 (3)	0.0552 (13)	
H19	1.1028	0.1787	0.6077	0.066*	
C20	0.9152 (5)	−0.0404 (3)	0.6468 (3)	0.0635 (14)	
H20	0.8651	−0.0968	0.6550	0.076*	
C21	1.0662 (5)	−0.0144 (3)	0.6794 (3)	0.0478 (12)	
C22	1.1335 (5)	0.0697 (3)	0.6623 (3)	0.0540 (13)	
H22	1.2337	0.0896	0.6813	0.065*	
C23	1.1428 (5)	−0.0742 (3)	0.7272 (3)	0.0526 (13)	
H23	1.0862	−0.1232	0.7427	0.063*	
C24	1.2871 (6)	−0.0651 (3)	0.7510 (3)	0.0541 (13)	
H24	1.3425	−0.0153	0.7359	0.065*	
C25	1.3682 (5)	−0.1241 (3)	0.7977 (3)	0.0504 (12)	
C26	1.3820 (5)	−0.2511 (3)	0.8750 (3)	0.0557 (13)	
H26	1.3346	−0.2974	0.8976	0.067*	
C27	1.3024 (5)	−0.1962 (3)	0.8305 (3)	0.0588 (14)	
H27	1.2016	−0.2080	0.8223	0.071*	
C28	1.5339 (5)	−0.2374 (3)	0.8861 (3)	0.0530 (13)	
C29	1.5983 (5)	−0.1674 (3)	0.8506 (3)	0.0638 (14)	
H29	1.6980	−0.1582	0.8551	0.077*	
C30	1.5184 (5)	−0.1116 (3)	0.8091 (3)	0.0590 (14)	
H30	1.5662	−0.0640	0.7879	0.071*	
C31	1.5453 (6)	−0.3641 (4)	0.9673 (4)	0.0896 (19)	
H31A	1.4932	−0.3382	1.0000	0.134*	
H31B	1.6189	−0.3914	0.9981	0.134*	
H31C	1.4788	−0.4104	0.9288	0.134*	
C32	1.7695 (5)	−0.2821 (4)	0.9372 (3)	0.0757 (16)	
H32A	1.7882	−0.2806	0.8870	0.114*	
H32B	1.8026	−0.3334	0.9602	0.114*	
H32C	1.8205	−0.2259	0.9696	0.114*	
N4	0.6240 (4)	0.4654 (2)	0.5851 (2)	0.0469 (10)	
N5	1.1525 (4)	−0.0005 (3)	0.9282 (2)	0.0664 (12)	
C33	0.5267 (5)	0.5205 (3)	0.5418 (2)	0.0505 (12)	
H33A	0.5790	0.5835	0.5455	0.061*	
H33B	0.4444	0.5213	0.5639	0.061*	
C34	0.5649 (5)	0.3890 (3)	0.6118 (3)	0.0534 (13)	
H34	0.4642	0.3724	0.6025	0.064*	
C35	0.7694 (5)	0.4882 (3)	0.5966 (3)	0.0673 (15)	
H35	0.8105	0.5396	0.5769	0.081*	
C36	0.8567 (5)	0.4360 (3)	0.6370 (3)	0.0631 (15)	
H36	0.9570	0.4528	0.6442	0.076*	
C37	0.6502 (5)	0.3360 (3)	0.6520 (3)	0.0515 (12)	
H37	0.6068	0.2834	0.6694	0.062*	
C38	0.8017 (5)	0.3593 (3)	0.6676 (2)	0.0423 (11)	
C39	0.8966 (5)	0.3074 (3)	0.7133 (3)	0.0477 (12)	
H39	0.9966	0.3300	0.7249	0.057*	
C40	0.8500 (5)	0.2291 (3)	0.7397 (3)	0.0473 (12)	
H40	0.7497	0.2073	0.7250	0.057*	
C41	0.9335 (5)	0.1731 (3)	0.7881 (3)	0.0465 (12)	
C42	0.8636 (5)	0.0878 (3)	0.8015 (3)	0.0591 (14)	
H42	0.7645	0.0684	0.7783	0.071*	
C43	1.0821 (5)	0.1978 (3)	0.8244 (3)	0.0525 (12)	
H43	1.1339	0.2541	0.8172	0.063*	
C44	1.1547 (5)	0.1424 (3)	0.8701 (3)	0.0509 (12)	
H44	1.2539	0.1622	0.8931	0.061*	
C45	1.0831 (5)	0.0564 (3)	0.8833 (3)	0.0490 (12)	
C46	0.9333 (5)	0.0314 (3)	0.8468 (3)	0.0626 (14)	
H46	0.8807	−0.0248	0.8538	0.075*	
C47	1.0752 (6)	−0.0900 (4)	0.9372 (3)	0.0810 (18)	
H47A	1.0364	−0.1261	0.8873	0.122*	
H47B	0.9969	−0.0828	0.9600	0.122*	
H47C	1.1409	−0.1207	0.9700	0.122*	
C48	1.3083 (5)	0.0216 (4)	0.9599 (3)	0.0752 (16)	
H48A	1.3555	0.0257	0.9185	0.113*	
H48B	1.3380	−0.0258	0.9900	0.113*	
H48C	1.3352	0.0795	0.9924	0.113*	

### Atomic displacement parameters (Å^2^)

**Table:**

	*U*^11^	*U*^22^	*U*^33^	*U*^12^	*U*^13^	*U*^23^
Br1	0.0648 (4)	0.0887 (5)	0.1078 (5)	0.0135 (3)	0.0259 (3)	0.0362 (4)
Br2	0.0549 (4)	0.0655 (4)	0.1300 (6)	0.0084 (3)	0.0344 (4)	0.0142 (4)
Br3	0.0541 (3)	0.0932 (5)	0.0877 (5)	0.0024 (3)	0.0060 (3)	0.0390 (4)
O1	0.131 (5)	0.157 (5)	0.102 (4)	0.054 (4)	0.012 (3)	0.014 (4)
C49	0.119 (6)	0.095 (5)	0.144 (8)	0.034 (5)	0.017 (5)	−0.029 (5)
C50	0.104 (5)	0.098 (5)	0.148 (7)	0.023 (4)	0.010 (5)	0.024 (5)
N1	0.052 (3)	0.049 (2)	0.051 (3)	0.014 (2)	0.004 (2)	0.010 (2)
N2	0.071 (3)	0.068 (3)	0.094 (4)	0.023 (3)	0.016 (3)	0.043 (3)
N3	0.055 (3)	0.072 (3)	0.078 (3)	0.017 (2)	0.008 (2)	0.030 (3)
N6	0.053 (3)	0.056 (3)	0.052 (3)	0.021 (2)	0.020 (2)	0.020 (2)
C1	0.056 (3)	0.048 (3)	0.049 (3)	0.017 (3)	0.004 (2)	0.003 (2)
C2	0.065 (4)	0.073 (4)	0.075 (4)	0.008 (3)	0.014 (3)	0.035 (3)
C3	0.052 (3)	0.078 (4)	0.073 (4)	0.009 (3)	0.005 (3)	0.028 (3)
C4	0.049 (3)	0.055 (3)	0.075 (4)	0.014 (3)	0.005 (3)	0.014 (3)
C5	0.056 (3)	0.052 (3)	0.086 (4)	0.013 (3)	0.014 (3)	0.019 (3)
C6	0.055 (3)	0.057 (3)	0.048 (3)	0.018 (3)	0.009 (2)	0.003 (3)
C7	0.060 (3)	0.062 (3)	0.048 (3)	0.016 (3)	0.009 (3)	0.007 (3)
C8	0.055 (3)	0.058 (3)	0.044 (3)	0.020 (3)	0.014 (2)	0.014 (2)
C9	0.049 (3)	0.077 (4)	0.076 (4)	0.013 (3)	0.016 (3)	0.029 (3)
C10	0.077 (4)	0.048 (3)	0.056 (3)	0.014 (3)	0.009 (3)	0.011 (3)
C11	0.065 (3)	0.059 (3)	0.061 (4)	0.013 (3)	0.000 (3)	0.008 (3)
C12	0.058 (3)	0.061 (3)	0.051 (3)	0.019 (3)	0.016 (3)	0.014 (3)
C13	0.066 (4)	0.071 (4)	0.090 (4)	0.020 (3)	0.030 (3)	0.037 (3)
C14	0.083 (4)	0.088 (5)	0.158 (7)	0.025 (4)	0.037 (4)	0.072 (4)
C15	0.074 (4)	0.094 (4)	0.078 (4)	0.029 (3)	0.004 (3)	0.025 (3)
C16	0.059 (3)	0.052 (3)	0.053 (3)	0.023 (3)	0.013 (3)	0.007 (2)
C17	0.063 (3)	0.058 (3)	0.050 (3)	0.022 (3)	0.020 (3)	0.012 (2)
C18	0.053 (3)	0.063 (3)	0.081 (4)	0.017 (3)	0.024 (3)	0.032 (3)
C19	0.060 (3)	0.052 (3)	0.059 (3)	0.013 (3)	0.020 (3)	0.018 (3)
C20	0.055 (3)	0.058 (3)	0.086 (4)	0.016 (3)	0.026 (3)	0.028 (3)
C21	0.056 (3)	0.050 (3)	0.047 (3)	0.024 (2)	0.020 (2)	0.009 (2)
C22	0.055 (3)	0.053 (3)	0.060 (3)	0.023 (3)	0.015 (3)	0.016 (3)
C23	0.060 (3)	0.054 (3)	0.053 (3)	0.022 (3)	0.022 (3)	0.015 (2)
C24	0.069 (4)	0.050 (3)	0.050 (3)	0.018 (3)	0.021 (3)	0.011 (2)
C25	0.056 (3)	0.047 (3)	0.050 (3)	0.016 (2)	0.012 (2)	0.009 (2)
C26	0.056 (3)	0.059 (3)	0.060 (3)	0.018 (3)	0.021 (3)	0.022 (3)
C27	0.052 (3)	0.073 (4)	0.060 (3)	0.024 (3)	0.020 (3)	0.013 (3)
C28	0.056 (3)	0.054 (3)	0.047 (3)	0.016 (3)	0.005 (2)	0.007 (3)
C29	0.047 (3)	0.068 (3)	0.070 (4)	0.005 (3)	0.004 (3)	0.019 (3)
C30	0.057 (3)	0.051 (3)	0.064 (4)	0.004 (3)	0.007 (3)	0.018 (3)
C31	0.092 (4)	0.090 (4)	0.102 (5)	0.036 (4)	0.028 (4)	0.052 (4)
C32	0.057 (3)	0.089 (4)	0.075 (4)	0.020 (3)	−0.002 (3)	0.014 (3)
N4	0.054 (3)	0.043 (2)	0.045 (2)	0.020 (2)	0.0061 (19)	0.0125 (19)
N5	0.057 (3)	0.070 (3)	0.077 (3)	0.025 (2)	0.010 (2)	0.042 (3)
C33	0.062 (3)	0.049 (3)	0.043 (3)	0.025 (2)	0.005 (2)	0.009 (2)
C34	0.045 (3)	0.061 (3)	0.056 (3)	0.013 (3)	0.011 (2)	0.022 (3)
C35	0.053 (3)	0.058 (3)	0.082 (4)	0.000 (3)	0.002 (3)	0.029 (3)
C36	0.043 (3)	0.055 (3)	0.086 (4)	0.004 (2)	0.004 (3)	0.032 (3)
C37	0.050 (3)	0.045 (3)	0.062 (3)	0.009 (2)	0.014 (3)	0.022 (2)
C38	0.044 (3)	0.040 (3)	0.041 (3)	0.014 (2)	0.004 (2)	0.004 (2)
C39	0.043 (3)	0.050 (3)	0.051 (3)	0.012 (2)	0.011 (2)	0.009 (2)
C40	0.042 (3)	0.055 (3)	0.047 (3)	0.015 (2)	0.011 (2)	0.010 (2)
C41	0.044 (3)	0.050 (3)	0.047 (3)	0.015 (2)	0.009 (2)	0.011 (2)
C42	0.043 (3)	0.062 (3)	0.068 (4)	0.005 (3)	0.007 (3)	0.024 (3)
C43	0.055 (3)	0.043 (3)	0.062 (3)	0.011 (2)	0.016 (3)	0.015 (2)
C44	0.043 (3)	0.055 (3)	0.052 (3)	0.017 (2)	0.001 (2)	0.013 (2)
C45	0.055 (3)	0.056 (3)	0.042 (3)	0.018 (3)	0.015 (2)	0.016 (2)
C46	0.054 (3)	0.057 (3)	0.081 (4)	0.013 (3)	0.016 (3)	0.035 (3)
C47	0.083 (4)	0.080 (4)	0.088 (5)	0.025 (3)	0.020 (3)	0.043 (3)
C48	0.065 (4)	0.080 (4)	0.075 (4)	0.025 (3)	−0.006 (3)	0.027 (3)

### Geometric parameters (Å, º)

**Table:**

O1—H1	0.8200	C21—C23	1.454 (6)
O1—C49	1.344 (7)	C22—H22	0.9300
C49—H49A	0.9700	C23—H23	0.9300
C49—H49B	0.9700	C23—C24	1.334 (6)
C49—C50	1.549 (10)	C24—H24	0.9300
C50—H50A	0.9600	C24—C25	1.457 (6)
C50—H50B	0.9600	C25—C27	1.387 (6)
C50—H50C	0.9600	C25—C30	1.391 (6)
N1—C3	1.336 (6)	C26—H26	0.9300
N1—C5	1.347 (6)	C26—C27	1.385 (6)
N1—C16	1.486 (6)	C26—C28	1.407 (6)
N2—C12	1.389 (6)	C27—H27	0.9300
N2—C14	1.428 (6)	C28—C29	1.390 (6)
N2—C15	1.438 (6)	C29—H29	0.9300
N3—C28	1.372 (6)	C29—C30	1.371 (6)
N3—C31	1.453 (6)	C30—H30	0.9300
N3—C32	1.458 (6)	C31—H31A	0.9600
N6—C17	1.487 (6)	C31—H31B	0.9600
N6—C18	1.339 (6)	C31—H31C	0.9600
N6—C19	1.345 (6)	C32—H32A	0.9600
C1—C2	1.403 (7)	C32—H32B	0.9600
C1—C4	1.390 (6)	C32—H32C	0.9600
C1—C6	1.451 (6)	N4—C33	1.476 (5)
C2—H2	0.9300	N4—C34	1.349 (5)
C2—C3	1.373 (7)	N4—C35	1.345 (6)
C3—H3	0.9300	N5—C45	1.363 (6)
C4—H4	0.9300	N5—C47	1.445 (6)
C4—C5	1.366 (7)	N5—C48	1.442 (6)
C5—H5	0.9300	C33—C33^i^	1.505 (8)
C6—H6	0.9300	C33—H33A	0.9700
C6—C7	1.315 (6)	C33—H33B	0.9700
C7—H7	0.9300	C34—H34	0.9300
C7—C8	1.459 (6)	C34—C37	1.360 (6)
C8—C9	1.386 (6)	C35—H35	0.9300
C8—C10	1.399 (6)	C35—C36	1.365 (6)
C9—H9	0.9300	C36—H36	0.9300
C9—C13	1.376 (7)	C36—C38	1.377 (6)
C10—H10	0.9300	C37—H37	0.9300
C10—C11	1.377 (6)	C37—C38	1.395 (6)
C11—H11	0.9300	C38—C39	1.447 (6)
C11—C12	1.394 (6)	C39—H39	0.9300
C12—C13	1.392 (6)	C39—C40	1.333 (6)
C13—H13	0.9300	C40—H40	0.9300
C14—H14A	0.9600	C40—C41	1.452 (6)
C14—H14B	0.9600	C41—C42	1.391 (6)
C14—H14C	0.9600	C41—C43	1.395 (6)
C15—H15A	0.9600	C42—H42	0.9300
C15—H15B	0.9600	C42—C46	1.363 (6)
C15—H15C	0.9600	C43—H43	0.9300
C16—H16A	0.9700	C43—C44	1.367 (6)
C16—H16B	0.9700	C44—H44	0.9300
C16—C17	1.495 (6)	C44—C45	1.406 (6)
C17—H17A	0.9700	C45—C46	1.405 (6)
C17—H17B	0.9700	C46—H46	0.9300
C18—H18	0.9300	C47—H47A	0.9600
C18—C20	1.359 (7)	C47—H47B	0.9600
C19—H19	0.9300	C47—H47C	0.9600
C19—C22	1.356 (6)	C48—H48A	0.9600
C20—H20	0.9300	C48—H48B	0.9600
C20—C21	1.404 (6)	C48—H48C	0.9600
C21—C22	1.393 (6)		
			
C49—O1—H1	109.5	C24—C23—H23	117.1
O1—C49—H49A	110.3	C23—C24—H24	116.2
O1—C49—H49B	110.3	C23—C24—C25	127.6 (5)
O1—C49—C50	106.9 (7)	C25—C24—H24	116.2
H49A—C49—H49B	108.6	C27—C25—C24	123.2 (4)
C50—C49—H49A	110.3	C27—C25—C30	116.7 (4)
C50—C49—H49B	110.3	C30—C25—C24	120.1 (4)
C49—C50—H50A	109.5	C27—C26—H26	119.7
C49—C50—H50B	109.5	C27—C26—C28	120.5 (5)
C49—C50—H50C	109.5	C28—C26—H26	119.7
H50A—C50—H50B	109.5	C25—C27—H27	119.0
H50A—C50—H50C	109.5	C26—C27—C25	122.1 (4)
H50B—C50—H50C	109.5	C26—C27—H27	119.0
C3—N1—C5	120.3 (4)	N3—C28—C26	121.0 (4)
C3—N1—C16	120.4 (4)	N3—C28—C29	122.0 (5)
C5—N1—C16	119.3 (4)	C29—C28—C26	117.1 (4)
C12—N2—C14	120.4 (4)	C28—C29—H29	119.2
C12—N2—C15	121.1 (5)	C30—C29—C28	121.6 (5)
C14—N2—C15	117.9 (4)	C30—C29—H29	119.2
C28—N3—C31	121.5 (4)	C25—C30—H30	119.0
C28—N3—C32	121.3 (4)	C29—C30—C25	122.0 (5)
C31—N3—C32	117.2 (4)	C29—C30—H30	119.0
C18—N6—C17	120.9 (4)	N3—C31—H31A	109.5
C18—N6—C19	119.6 (4)	N3—C31—H31B	109.5
C19—N6—C17	119.6 (4)	N3—C31—H31C	109.5
C2—C1—C6	124.3 (5)	H31A—C31—H31B	109.5
C4—C1—C2	115.6 (4)	H31A—C31—H31C	109.5
C4—C1—C6	120.1 (4)	H31B—C31—H31C	109.5
C1—C2—H2	119.1	N3—C32—H32A	109.5
C3—C2—C1	121.7 (5)	N3—C32—H32B	109.5
C3—C2—H2	119.1	N3—C32—H32C	109.5
N1—C3—C2	120.1 (5)	H32A—C32—H32B	109.5
N1—C3—H3	119.9	H32A—C32—H32C	109.5
C2—C3—H3	119.9	H32B—C32—H32C	109.5
C1—C4—H4	119.4	C34—N4—C33	119.0 (4)
C5—C4—C1	121.1 (5)	C35—N4—C33	121.4 (4)
C5—C4—H4	119.4	C35—N4—C34	119.6 (4)
N1—C5—C4	121.2 (5)	C45—N5—C47	120.8 (4)
N1—C5—H5	119.4	C45—N5—C48	120.9 (4)
C4—C5—H5	119.4	C48—N5—C47	117.8 (4)
C1—C6—H6	117.3	N4—C33—C33^i^	109.5 (4)
C7—C6—C1	125.5 (5)	N4—C33—H33A	109.8
C7—C6—H6	117.3	N4—C33—H33B	109.8
C6—C7—H7	116.4	C33^i^—C33—H33A	109.8
C6—C7—C8	127.3 (5)	C33^i^—C33—H33B	109.8
C8—C7—H7	116.4	H33A—C33—H33B	108.2
C9—C8—C7	120.3 (4)	N4—C34—H34	119.4
C9—C8—C10	116.6 (4)	N4—C34—C37	121.1 (4)
C10—C8—C7	123.1 (5)	C37—C34—H34	119.4
C8—C9—H9	119.0	N4—C35—H35	119.9
C13—C9—C8	121.9 (5)	N4—C35—C36	120.1 (5)
C13—C9—H9	119.0	C36—C35—H35	119.9
C8—C10—H10	119.3	C35—C36—H36	118.8
C11—C10—C8	121.5 (5)	C35—C36—C38	122.3 (4)
C11—C10—H10	119.3	C38—C36—H36	118.8
C10—C11—H11	119.1	C34—C37—H37	119.5
C10—C11—C12	121.8 (5)	C34—C37—C38	121.0 (4)
C12—C11—H11	119.1	C38—C37—H37	119.5
N2—C12—C11	121.5 (5)	C36—C38—C37	115.8 (4)
N2—C12—C13	122.1 (5)	C36—C38—C39	121.4 (4)
C13—C12—C11	116.4 (5)	C37—C38—C39	122.8 (4)
C9—C13—C12	121.8 (5)	C38—C39—H39	117.9
C9—C13—H13	119.1	C40—C39—C38	124.2 (4)
C12—C13—H13	119.1	C40—C39—H39	117.9
N2—C14—H14A	109.5	C39—C40—H40	115.4
N2—C14—H14B	109.5	C39—C40—C41	129.2 (4)
N2—C14—H14C	109.5	C41—C40—H40	115.4
H14A—C14—H14B	109.5	C42—C41—C40	119.4 (4)
H14A—C14—H14C	109.5	C42—C41—C43	115.4 (4)
H14B—C14—H14C	109.5	C43—C41—C40	125.2 (4)
N2—C15—H15A	109.5	C41—C42—H42	118.4
N2—C15—H15B	109.5	C46—C42—C41	123.2 (4)
N2—C15—H15C	109.5	C46—C42—H42	118.4
H15A—C15—H15B	109.5	C41—C43—H43	118.7
H15A—C15—H15C	109.5	C44—C43—C41	122.6 (4)
H15B—C15—H15C	109.5	C44—C43—H43	118.7
N1—C16—H16A	109.8	C43—C44—H44	119.2
N1—C16—H16B	109.8	C43—C44—C45	121.6 (4)
N1—C16—C17	109.5 (4)	C45—C44—H44	119.2
H16A—C16—H16B	108.2	N5—C45—C44	123.0 (4)
C17—C16—H16A	109.8	N5—C45—C46	121.1 (4)
C17—C16—H16B	109.8	C46—C45—C44	115.9 (4)
C16—C17—H17A	109.8	C42—C46—C45	121.3 (5)
C16—C17—H17B	109.8	C42—C46—H46	119.4
H17A—C17—H17B	108.3	C45—C46—H46	119.4
C20—C18—H18	119.4	N5—C47—H47A	109.5
C22—C19—H19	119.3	N5—C47—H47B	109.5
C18—C20—H20	119.5	N5—C47—H47C	109.5
C18—C20—C21	121.0 (5)	H47A—C47—H47B	109.5
C21—C20—H20	119.5	H47A—C47—H47C	109.5
C20—C21—C23	120.2 (4)	H47B—C47—H47C	109.5
C22—C21—C20	115.8 (4)	N5—C48—H48A	109.5
C22—C21—C23	124.0 (4)	N5—C48—H48B	109.5
C19—C22—C21	121.0 (5)	N5—C48—H48C	109.5
C19—C22—H22	119.5	H48A—C48—H48B	109.5
C21—C22—H22	119.5	H48A—C48—H48C	109.5
C21—C23—H23	117.1	H48B—C48—H48C	109.5
C24—C23—C21	125.9 (5)		
			
N1—C16—C17—N6	−174.9 (4)	C23—C21—C22—C19	−178.8 (5)
N2—C12—C13—C9	178.4 (5)	C23—C24—C25—C27	−5.8 (8)
N3—C28—C29—C30	−178.2 (5)	C23—C24—C25—C30	171.8 (5)
N6—C18—C20—C21	0.3 (8)	C24—C25—C27—C26	−179.8 (5)
N6—C19—C22—C21	0.2 (7)	C24—C25—C30—C29	−177.8 (5)
C1—C2—C3—N1	1.1 (9)	C26—C28—C29—C30	2.6 (8)
C1—C4—C5—N1	0.1 (8)	C27—C25—C30—C29	−0.1 (7)
C1—C6—C7—C8	−177.8 (5)	C27—C26—C28—N3	−179.4 (5)
C2—C1—C4—C5	0.2 (7)	C27—C26—C28—C29	−0.2 (7)
C2—C1—C6—C7	−16.6 (8)	C28—C26—C27—C25	−2.4 (8)
C3—N1—C5—C4	0.2 (8)	C28—C29—C30—C25	−2.5 (8)
C3—N1—C16—C17	102.0 (5)	C30—C25—C27—C26	2.5 (7)
C4—C1—C2—C3	−0.8 (8)	C31—N3—C28—C26	−1.8 (8)
C4—C1—C6—C7	164.6 (5)	C31—N3—C28—C29	179.1 (5)
C5—N1—C3—C2	−0.7 (8)	C32—N3—C28—C26	176.0 (5)
C5—N1—C16—C17	−77.7 (5)	C32—N3—C28—C29	−3.1 (8)
C6—C1—C2—C3	−179.7 (5)	N4—C34—C37—C38	0.6 (7)
C6—C1—C4—C5	179.1 (5)	N4—C35—C36—C38	−0.2 (8)
C6—C7—C8—C9	169.2 (5)	N5—C45—C46—C42	−179.6 (5)
C6—C7—C8—C10	−10.2 (8)	C33—N4—C34—C37	180.0 (4)
C7—C8—C9—C13	−178.9 (5)	C33—N4—C35—C36	179.9 (4)
C7—C8—C10—C11	180.0 (5)	C34—N4—C33—C33^i^	−83.7 (6)
C8—C9—C13—C12	−0.3 (8)	C34—N4—C35—C36	−2.0 (8)
C8—C10—C11—C12	−1.8 (8)	C34—C37—C38—C36	−2.7 (7)
C9—C8—C10—C11	0.6 (7)	C34—C37—C38—C39	176.9 (4)
C10—C8—C9—C13	0.5 (8)	C35—N4—C33—C33^i^	94.4 (6)
C10—C11—C12—N2	−177.4 (5)	C35—N4—C34—C37	1.8 (7)
C10—C11—C12—C13	2.0 (8)	C35—C36—C38—C37	2.5 (8)
C11—C12—C13—C9	−0.9 (8)	C35—C36—C38—C39	−177.1 (5)
C14—N2—C12—C11	−178.2 (6)	C36—C38—C39—C40	−175.0 (5)
C14—N2—C12—C13	2.5 (8)	C37—C38—C39—C40	5.4 (7)
C15—N2—C12—C11	−6.9 (8)	C38—C39—C40—C41	−177.4 (5)
C15—N2—C12—C13	173.8 (5)	C39—C40—C41—C42	−172.5 (5)
C16—N1—C3—C2	179.5 (5)	C39—C40—C41—C43	8.1 (8)
C16—N1—C5—C4	179.9 (4)	C40—C41—C42—C46	−179.2 (5)
C17—N6—C18—C20	−178.3 (5)	C40—C41—C43—C44	179.3 (5)
C17—N6—C19—C22	178.1 (4)	C41—C42—C46—C45	−0.3 (9)
C18—N6—C17—C16	−89.0 (5)	C41—C43—C44—C45	0.1 (8)
C18—N6—C19—C22	−1.9 (7)	C42—C41—C43—C44	−0.2 (7)
C18—C20—C21—C22	−1.9 (7)	C43—C41—C42—C46	0.3 (8)
C18—C20—C21—C23	178.5 (5)	C43—C44—C45—N5	179.6 (5)
C19—N6—C17—C16	91.0 (5)	C43—C44—C45—C46	−0.1 (7)
C19—N6—C18—C20	1.6 (7)	C44—C45—C46—C42	0.2 (8)
C20—C21—C22—C19	1.7 (7)	C47—N5—C45—C44	−177.2 (5)
C20—C21—C23—C24	168.4 (5)	C47—N5—C45—C46	2.5 (8)
C21—C23—C24—C25	−179.0 (4)	C48—N5—C45—C44	−5.3 (8)
C22—C21—C23—C24	−11.1 (8)	C48—N5—C45—C46	174.5 (5)

Symmetry code: (i) −*x*+1, −*y*+1, −*z*+1.

### Hydrogen-bond geometry (Å, º)

**Table:**

*D*—H···*A*	*D*—H	H···*A*	*D*···*A*	*D*—H···*A*
O1—H1···Br1^ii^	0.82	2.50	3.310 (6)	171
C16—H16*B*···Br2	0.97	2.92	3.841 (5)	158
C19—H19···Br3	0.93	2.87	3.765 (5)	162
C34—H34···Br3^iii^	0.93	2.85	3.761 (5)	167

Symmetry codes: (ii) −*x*+2, −*y*+1, −*z*+1; (iii) *x*−1, *y*, *z*.
